# Expression and Functions of CreD, an Inner Membrane Protein in *Stenotrophomonas maltophilia*

**DOI:** 10.1371/journal.pone.0145009

**Published:** 2015-12-23

**Authors:** Hsin-Hui Huang, Yi-Tsung Lin, Wei-Ching Chen, Yi-Wei Huang, Shiang-Jiuun Chen, Tsuey-Ching Yang

**Affiliations:** 1 Department of Biotechnology and Laboratory Science in Medicine, National Yang-Ming University, Taipei, Taiwan; 2 Division of Infectious Diseases, Department of Medicine, Taipei Veterans General Hospital, Taipei, Taiwan; 3 School of Medicine, National Yang-Ming University, Taipei, Taiwan; 4 Department of Life Science, Institute of Ecology and Evolutionary Biology and TechComm-5, College of Life Science, National Taiwan University, Taipei, Taiwan; Niels Bohr Institute, DENMARK

## Abstract

CreBC is a highly conserved two-component regulatory system (TCS) in several gram-negative bacteria, including *Escherichia coli*, *Aeromonas* spp., *Pseudomonas aeruginosa*, and *Stenotrophomonas maltophilia*. *CreD* is a conserved gene that encodes a predicted inner-membrane protein and is located near the *creBC* loci. Activation of CreBC increases *creD* expression; therefore, *creD* expression is generally used as a measure of CreBC activation in *E*. *coli*, *Aeromonas* spp., and *P*. *aeruginosa* systems. In this article, we aim to elucidate the expression of *creD* and further to investigate its functions in *S*. *maltophilia*. In spite of a short intergenic region of 81 bp between *creBC* and *creD*, *creD* is expressed separately from the adjacent *creBC* operon and from a promoter immediately upstream of *creD* (*P*_*creD*_) in *S*. *maltophilia*. We found that the promoter activity of *P*_*creD*_ is negatively regulated by the *creBC* TCS, positively regulated by the bacterial culture density, and not affected by β-lactams. Furthermore, *creD* expression is not significantly altered in the presence of the phosphor-mimic variant of CreB, CreB(D55E), which mimics activated CreB. The functions of CreD of *S*. *maltophilia* were assessed by comparison among the following: wild-type KJ; the *creD* isogenic mutant, KJΔCreD; and the complementary strain, KJΔCreD(pCreD). The mutant lacking *creD* had cell division defects and aberrations in cell envelope integrity, which then triggered the σ^E^-mediated envelope stress response. Thus, the results indicated that CreD plays a critical role in the maintenance of envelope integrity.

## Introduction

Two-component regulatory systems (TCSs) are basic stimulus-response coupling mechanisms that allow organisms to sense and respond to changes in environmental conditions. TCSs consist of an inner membrane-spanning sensor histidine kinase (HK) and a cognate cytoplasmic response regulator protein (RR) [1]. Extracellular stimuli are sensed by HK, and HK is modulated by autophosphorylation. The HK transfers a phosphoryl group to the RR, which activates or represses the expression of an array of genes called the TCS regulon. To characterize a TCS, both the stimulus and response must be considered. Therefore, TCS activation is commonly evaluated by assessing gene expression of the responsive regulon. Some TCSs are highly conserved in different microorganisms; for example, the PhoP/PhoQ system is found in *Escherichia coli*, *Salmonella enterica*, and *Pseudomonas aeruginosa* [2]. These TCS homologues in different microorganisms likely share same or similar activation stimuli and responsive regulons.

CreBC/BlrAB TCS, consisting of a sensor kinase (CreC/BlrB) and a response regulator (CreB/BlrA), exists in many gram-negative bacteria, such as *E*. *coli*, *Aeromonas* spp., and *P*. *aeruginosa* (S1 Fig). In spite of the high conservation of the CreBC TCS in different gram-negative bacteria, the conditions known to activate CreBC vary in different microorganisms. In *E*. *coli*, *creBC* is activated under fermentative growth conditions using glycolytic carbon sources and under aerobic conditions with low-molecular-weight fermentation products as substrates, such as formate or pyruvate [3]. However, the activation of *blrAB* and *creBC* in *Aeromonas* spp. and *P*. *aeruginosa* is triggered by the loss of function of penicillin-binding protein 4 (PBP4) [4, 5]. Collectively, CreBC/BlrAB responds to metabolic signals or peptidoglycan stress. The CreBC/BlrAB regulon members in *E*. *coli*, *Aeromonas* spp., and *P*. *aeruginosa* have been previously reported [6–8]. A common tightly controlled *cre* regulon gene is *creD*, which is located near *creBC* and is part of the *creABCD* cluster of *E*. *coli*, the *blrABD* cluster of *Aeromonas* spp., and the *creBCD* cluster of *P*. *aeruginosa* (S1 Fig). *CreD* expression is positively regulated by activated CreBC in *E*. *coli* and *P*. *aeruginosa* [3, 5] and by activated BlrAB in *Aeromonas* spp. [9]. A *cre/blr* tag sequence, TTCACN_6_TTCAC, is upstream of the *creD* gene and is critical for binding activated CreB/BlrA transcription regulators [6]. Therefore, increased expression of *creD* is an indicator for *creBC* TCS activation in *E*. *coli*, *Aeromonas* spp., and *P*. *aeruginosa* systems. However, there is little published paper addressing the physiological function of CreD.

*Stenotrophomonas maltophilia* is a gram-negative, genetically versatile, and environmentally ubiquitous bacterial species [10]. *S*. *maltophilia* can survive in a variety of animal and plant hosts and environmental niches. Moreover, *S*. *maltophilia* causes opportunistic infections, especially in patients with cystic fibrosis or who are immune compromised. For survival in different environmental niches, *S*. *maltophilia* has developed an array of TCSs to coordinate the expression of genes involved in adaptation. Based on its sequenced genome [11], *S*. *maltophilia* harbors at least 43 sets of TCSs. Of these, only SmeSR and CreBC have been investigated [12, 13]. However, little is understood about stimuli that activate these TCSs and gene regulons that are regulated by these TCSs.

**Fig 1:**
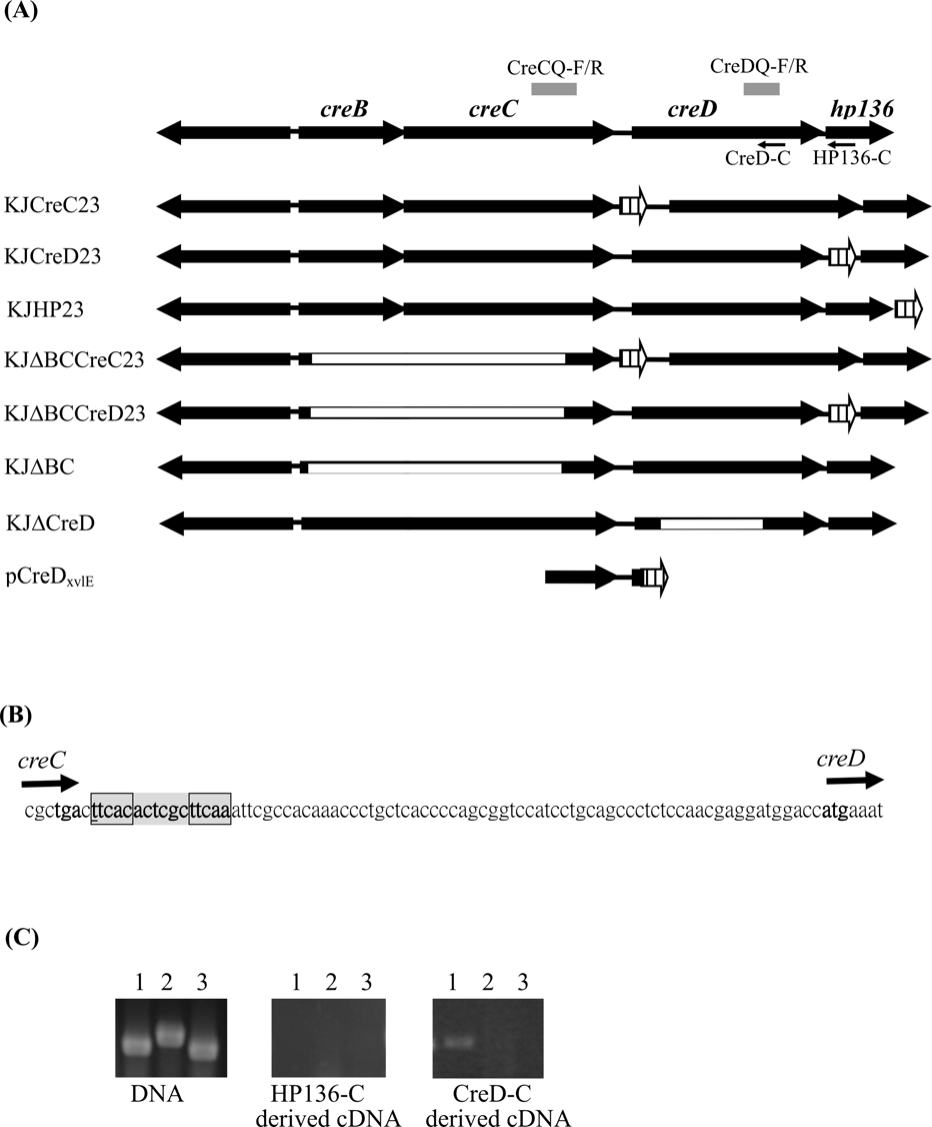
Schematic organization and operon verification of the *creBCD-hp136* cluster of *S*. *maltophilia* KJ. (A) Genomic organization of the *creBCD-hp136* cluster and the structure of chromosomal *xylE*-transcription fusion constructs, mutants, and recombinant plasmids. The *creBC* operon forms a two-component regulatory system. *CreD* encodes a putative inner membrane protein. The small arrows indicate the primer HP136-C and CreD-C for the reverse transcription. The gray bar indicates the PCR amplicons using the CreDQ-F/R and CreCQ-F/R as the primer sets. The orientation of gene is indicated by the arrow. The white box indicates the deleted region for each deletion mutant construct. The crosshatched arrows represent the *xylE* cassette. (B) The 81-bp DNA sequence of intergenic region between *creC* and *creD*. A homologue of so-called *cre/blr* tag sequence (TTCACnnnnnnTTCAA) is marked in gray, at around -80 to -65 bp relative to the start codon of *creD*. (C) Agarose gel electrophoresis of reverse transcriptase-PCR (RT-PCR). The cDNAs of *S*. *maltophilia* KJ were obtained by RT-PCR using the primers HP136-C and CreD-C respectively, and then PCR was performed using different primer sets. The *S*. *maltophilia* KJ chromosome DNA was used as a control for the primers reliability. Lane 1, primers CreDQ-F and CreDQ-R; Lane 2, primers CreCQ-F and CreCQ-R; Lane 3, primers SmeXQ-F and SmeXQ-R. *SmeX* gene, which is intrinsically unexpressed in strain KJ, is used as a control for the DNA contamination during cDNA preparation.

Like *E*. *coli*, *Aeromonas* spp., and *P*. *aeruginosa*, *S*. *maltophilia* harbors the CreBC TCS. The *creD* homologue is conserved and located downstream of *creBC* in *S*. *maltophilia* (Fig 1A). Furthermore, a putative *cre/blr* tag sequence homologue, TTCACACTCGCTTCAA, is located around -80 to -65 bp relative to the start codon of *creD* (Fig 1B). These observations suggest that the regulatory circuit of CreBC-CreD in *S*. *maltophilia* is similar to that in *E*. *coli*, *Aeromonas* spp., and *P*. *aeruginosa*. Nevertheless, we recently observed that the *creD* transcript has a 3.83 ± 0.33-fold increase when *creBC* is inactivated [14], which suggests that the regulatory circuit of *creD* expression in *S*. *maltophilia* is distinct and perhaps more complicated than that in *E*. *coli*, *Aeromonas* spp., and *P*. *aeruginosa*. In this article, we aim to further elucidate the factors that regulate *creD* expression and the physiological functions of CreD in *S*. *maltophilia*.

## Materials and Methods

### Bacterial strains and culture conditions

A complete list of strains, plasmids, and primers used in this study is shown in S1 Table *S*. *maltophilia* KJ served as the parental wild-type strain [15]. Cells were grown at 37°C in Luria-Bertani (LB) broth or Mueller-Hinton (MH) medium unless specified otherwise.

### Construction of chromosomal *xylE*-transcription fusion constructs KJCreC23, KJCreD23, KJHP23, KJΔBCCreC23, and KJΔBCCreD23

The chromosomal *creC-xylE*, *creD-xylE*, and *hp136-xylE* transcription fusion constructs were constructed by double crossover homologous recombination. Recombinant plasmids pCreC23, pCreD23, and pHP23 were prepared as follows: two DNA fragments containing the upstream and downstream region of the inserted site were obtained by PCR using specifically designed primer sets and subsequently cloned into pEX18Tc. A *xylE* cassette retrieved from pTxylE [15] was inserted between the two DNA fragments, yielding plasmids pCreC23, pCreD23, and pHP23. The primer sets used were CreC23N-F/CreC23N-R and CreC23C-F/CreC23C-R for pCreC23, CreD23N-F/CreD23N-R and CreD23C-F/CreD23C-R for pCreD23, and HP23N-F/HP23N-R and HP23C-F/HP23C-R for pHP23 (S1 Table). Plasmid mobilization and *xylE*-transcription fusion construct selection were performed as described previously [16]. The *xylE* gene in these recombinant plasmids was inserted into the targeted insertion site without disrupting any gene, generating chromosomal transcription fusion constructs KJCreC23, KJCreD23, and KJHP23 (Fig 1). The expression of *xylE* in these constructs represents expression of *creC*, *creD*, and *hp136*, respectively. The KJΔBCCreC23 and KJΔBCCreD23 constructs were obtained by inserting *xylE* downstream of the *creC* and *creD* genes of KJΔBC, respectively, through the same procedure.

### Quantitative real-time PCR (qRT-PCR)

Total RNA was isolated from exponential-growth bacterial cultures with the Pure Link^TM^ Total RNA Purification System (Invitrogen, Carlsbad, CA, USA) and RNase-free DNase (Invitrogen, Carlsbad, CA, USA) as described previously [16]. cDNA were synthesized from total RNA by using the MMLV Reverse Transcriptase 1^st^ Strand cDNA Synthesis kit (Epicentre Biotechnologies, Taiwan). QRT-PCR was performed with appropriate primer sets (S1 Table), cDNA, and the Smart Quant Green Master Mix (Protech Technology Enterprise Co., Ltd.), using a programmed ABI Prism 7000 Sequence Detection System (PE Applied Biosystems). The 16S rRNA gene was used as the normalizing gene. For relative gene expression analysis, a comparative cycle threshold method (*ΔΔCt*) was used [17].

### Construction of transcription fusion plasmid pCreD_xylE_

The 472-bp DNA fragment upstream of *creD* was obtained by PCR using primers CreD5-F and CreD5-R (S1 Table). The PCR amplicon was ligated into the promoter-less *xylE* reporter plasmid pRKXylE [18], generating pCreD_xylE_.

### Catechol 2,3-dioxygenase (C23O) activity assay

Catechol-2,3-dioxygenase is encoded by the *xylE* gene and its activity can be measured as the rate of increase in *A*_*375nm*_ following the addition of 100 mM catechol, as described previously [19]. The rate of hydrolysis is calculated by using 44,000 M^-1^·cm^-1^ as the extinction coefficient. One unit of enzyme activity (Uc) is defined as the amount of enzyme that converts 1 nmol of substrate per minute. The specific activity is expressed as Uc/OD_450 nm_.

### Site-directed mutagenesis of *creB* gene, yielding *creB(D55E)*

To generate a *creB(D55E)* allele in which amino acid 55 in CreB is switched from aspartate to glutamate, we used site-directed mutagenesis by primer extension PCR. Two PCR amplicons were obtained by PCR using the primer pairs CreB-F/CreB(D/E)N-R and CreB(D/E)C-F/CreB-R (S1 Table). The mutated nucleotide was introduced into the primers of CreB(D/E)N-R and CreB(D/E)C-F. Two PCR amplicons were mixed with the primer pair CreB-F/CreB-R for a second round of PCR. We checked the mutated *creB(D55E)* allele by DNA sequencing.

### Overexpression of *creB(D55E)* by a fusaric acid-inducible system

Our previous study described a *fuaABC* operon whose expression is inducible by fusaric acid [20]. Herein, we utilized the *fuaABC* operon to develop a fusaric acid-inducible overexpression system in *S*. *maltophilia*. First, vector pYW2 was constructed for cloning *creB(D55E)*. Two PCR amplicons of 382 bp and 370 bp, corresponding to the C-terminus of *fuaC* gene and downstream of the *fuaC* gene, respectively, were obtained by PCR, using primer sets YW2N-F/YW2N-R and YW2C-F/YW2C-R (S1 Table), respectively. The two PCR amplicons were sequentially cloned into pEX18Tc, resulting in plasmid pYW2. The *creB(D55E)* allele was cloned into pYW2 by inserting *creB(D55E)* between the two PCR amplicons, yielding pYW2CreB(D55E). Plasmid pYW2CreB(D55E) was mobilized from *E*. *coli* S17-1 into KJCreD23 by conjugation, and the correct double-crossover mutant, KJCreD23Fua::CreB(D55E), was selected and checked as previously described [16]. In strain KJCreD23Fua::CreB(D55E), the *creB(D55E)* allele was inserted downstream of the *fuaABC* operon without disruption of any gene, and the orientation of the *creB(D55E)* allele coincided with the transcription of the *fuaABC* operon.

### Construction of the deletion mutant KJΔCreD

The intact *creD* gene was amplified using the primers CreD-F and CreD-R (S1 Table) and cloned into pRK415 and pEX18Tc, yielding pCreD and pEXCreD, respectively. Plasmid pEXCreD was digested by StuI and then self-ligated to generate pΔCreD, in which the internal 519-bp StuI-StuI fragment of *creD* was deleted. Plasmid pΔCreD mobilization and mutant KJΔCreD selection were performed as described previously [16]. The correctness of deletion mutants was confirmed by PCR and DNA sequencing.

### Scanning electron microscopy (SEM)

The bacterial strains tested were grown to an OD_450nm_ of 1.0 and collected by centrifugation. Cells were washed three times with PBS, pH 7.4, and pre-fixed with 2.5% glutaraldehyde in phosphate buffer (0.1 M, pH 7.4) on glass coverslips. After the cells were fixed, they were washed, post-fixed with 1% osmium tetraoxide (OsO_4_), dehydrated by treatment with a graded ethanol series, dried to the critical point, and coated with gold particles. Then, the samples were examined using a high-resolution FEI Inspect S scanning electron microscope.

### Sodium dodecyl sulfate (SDS) survival analysis

Overnight cultures of the tested strains were diluted to an *A*_450nm_ of 0.15 with LB broth. This was followed by another 15-h culture, following which the cell cultures were adjusted to an *A*_450nm_ of 1.0 with the same broth. The cells were then treated with 0.01% SDS or were not treated. CFU analysis was performed after 5 min of incubation without shaking. The percentage of survival was defined as the CFU ratio of the SDS-additive group to the SDS-free counterpart.

### 1-N-phenylanphthylamine (NPN) uptake assay

Overnight cultures were subcultured into fresh LB broth and grown for 15 h. The cells were centrifuged and washed with 5 mM HEPES buffer (pH 7.2), and the optical density of the suspension was adjusted to an OD_450nm_ of 0.5 with the same buffer. Aliquots (100 μl) of the cell suspension were pipetted into 96-well microtiter plates, and NPN was then added at a final concentration of 15 μM. Fluorescence was monitored after 5-min incubation from three parallel wells per sample with a fluorescence spectrophotometer at excitation and emission wavelengths of 355 nm and 402 nm, respectively.

### Vancomycin susceptibility test

Susceptibility to vancomycin was assessed by E-test according to the manufacturer’s instructions. E-test strips were obtained from AB Biodisk (Solna, Sweden) and stored at −20°C until the tests were performed. The concentrations used ranged from 0.016 to 256 μg/ml for vancomycin. The MH plate was streaked with a cotton swab soaked in *S*. *maltophilia* cell suspension (10^7^ cells/ml), and an E-test strip was applied. The plates were incubated at 37°C, and the results were read after 24 h.

## Results

### Analysis of *creBCD* cluster in *S*. *maltophilia*

Fig 1A shows the genetic organization of *creD* in *S*. *maltophilia* KJ cells. There is an 81-bp intergenic region (IG) between *creC* and *creD*. *Hp136*, ten base pairs downstream of *creD*, encodes a predicted 136-aa hypothetical protein (HP136) (Fig 1A). The genetic organization of the four genes strongly suggests that *creB*, *creC*, *creD*, and *hp136* genes are organized into an operon.

**Fig 2:**
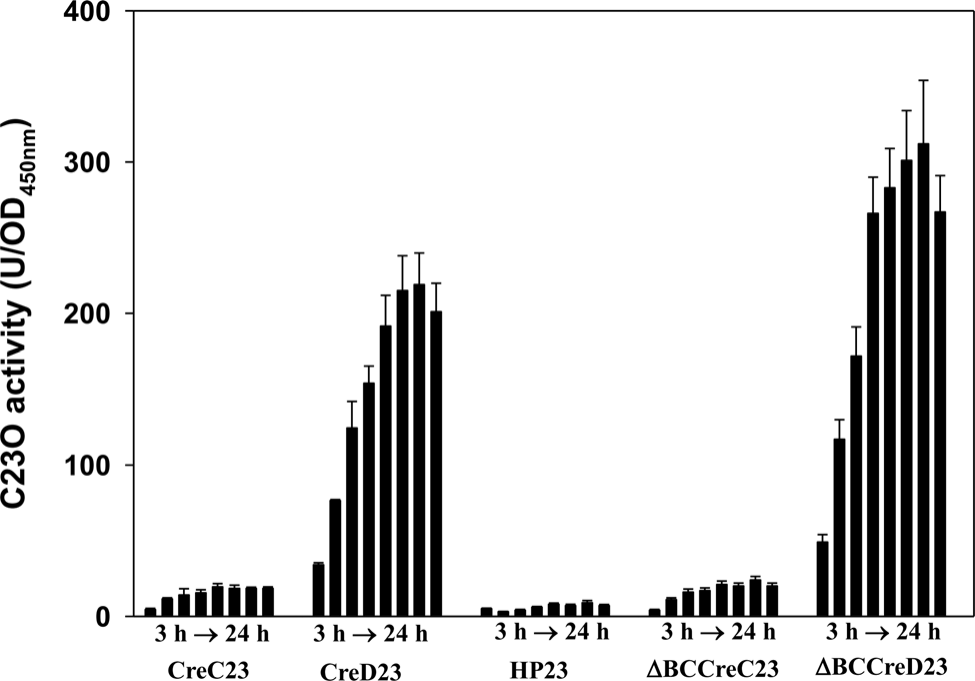
The C23O activity expressed by the chromosomal *xylE-*transcription fusion constructs of *S*. *maltophilia* KJ. Overnight cultures of *S*. *maltophilia* strains assayed were inoculated into the fresh LB to the *A*_450_ of 0.15. Cells were grown aerobically, and the *A*_450_ and C23O activity were measured every 3 h.

We used a chromosomal *in situ xylE*-transcription fusion assay and reverse transcription-PCR to verify the possibility of *creBCD* or *creBCD-hp136* operons. The *xylE* gene was inserted downstream of *creC*, *creD*, and *hp136* genes, yielding three chromosomal *xylE-*transcription fusion constructs, KJCreC23, KJCreD23, and KJHP23, respectively (Fig 1A). The C23O activities detected from KJCreC23, KJCreD23, and KJHP23 represent the amount of *creC*, *creD*, and *hp136* transcripts, respectively. Additionally, C23O activities reflect the promoter activity of *P*_*creB*_, *P*_*creD*_ and *P*_*hp136*_, if present, and could identify either *creBCD* or *creBCD-hp136* operons. The C23O activities determined from KJCreC23, KJCreD23, and KJHP23 cells are shown in Fig 2. The C23O expression in KJCreD23 increased with growth, KJCreC23 exhibited a moderate-level of C23O expression, and KJHP23 had no significant C23O activity. These results suggest that *creBCDsm-hp136* is not an operon, that the promoter of *creBC* TCS is intrinsically moderately active, and that *creD* has its own promoter, which is active in the laboratory cultured condition.

Primers CreD-C and HP136-C, which target the internal sequences of *creD* and *hp136*, respectively, were used to produce cDNAs from total RNA (Fig 1A, S1 Table). The cDNA was PCR amplified using primer sets CreCQ-F/R and CreDQ-F/R (Fig 1A, S1 Table), which target the *creC* and *creD* genes, respectively. The PCR amplicons with an expected size of 275 and 226 bps were observed only if the *creBCD* or *creBCD-hp136* transcripts were present. We did not detect the expected PCR amplicons in the HP136-C-derived cDNA group (Fig 1C), further ruling out the possibility of a *creBCD-hp136* operon. Furthermore, we did not detect the 275-bp amplicon in the CreD-C-derived cDNA (Fig 1C), indicating that no detectable *creBCD* transcript exists in KJ cells. The results of the chromosomal *xylE*-transcription fusion assay and RT-PCR indicated that *creBCD* is not an operon and that the promoter of *creD* in wild-type KJ cells is active in the laboratory cultured condition.

### CreBC TCS negatively regulates the promoter activity of *creD* (*P*_*creD*_)

To elucidate whether *creD* is regulated by the *creBC* TCS, a plasmid-borne promoter transcription fusion, pCreD_xylE_ (Fig 1A), was introduced into wild-type KJ and KJΔBC [14] and the C23O activity was measured. To our surprise, the C23O activity in KJ(pCreD_xylE_) was lower and did not increase with growth (S2 Fig). The inconsistency in results of the plasmid transcription fusion construct (KJ(pCreD_xylE_)) and the chromosomal transcription fusion construct (KJCreD23) indicates that there are some underlying regulatory mechanisms involved in the promoter activity of the *creD* gene, which requires further elucidation. To verify which assay best reflects *creD* expression, the *creD* transcript of wild-type KJ was determined by qRT-PCR. The *creD* transcript in KJ cells after 21 h of growth increased approximately 5.3 fold compared to the transcript levels after 6 h of growth, which is consistent with the results of KJCreD23. Therefore using the plasmid-borne promoter assay to assess the promoter activity of *creD* is infeasible. To elucidate the underlying regulatory mechanism in the *creBCD* cluster, we constructed four chromosomal *xylE-*transcription fusion constructs: KJCreC23, KJCreD23, KJΔBCCreC23, and KJΔBCCreD23 (Fig 1A).

The growth and C23O activities of these chromosomal *xylE*-transcription fusion constructs were monitored every 3 h. We made several conclusions by comparing the C23O activities among different pairwise combinations (Fig 2). First, the deletion of *creBC* did not affect *creBC* expression (KJCreC23 vs. KJΔBCCreC23), indicating that *creBC* TCS does not autoregulate. In addition, the CreBC TCS negatively regulated the expression of *creD*, as evidenced by comparing the C23O activities of strains KJΔBCCreD23 and KJCreD23. Finally, the *P*_*creD*_ activity was higher than *P*_*creBC*_, and both promoter activities increased with culture density.

### *P*_*creD*_ activity is regulated by bacterial culture density

**Fig 3:**
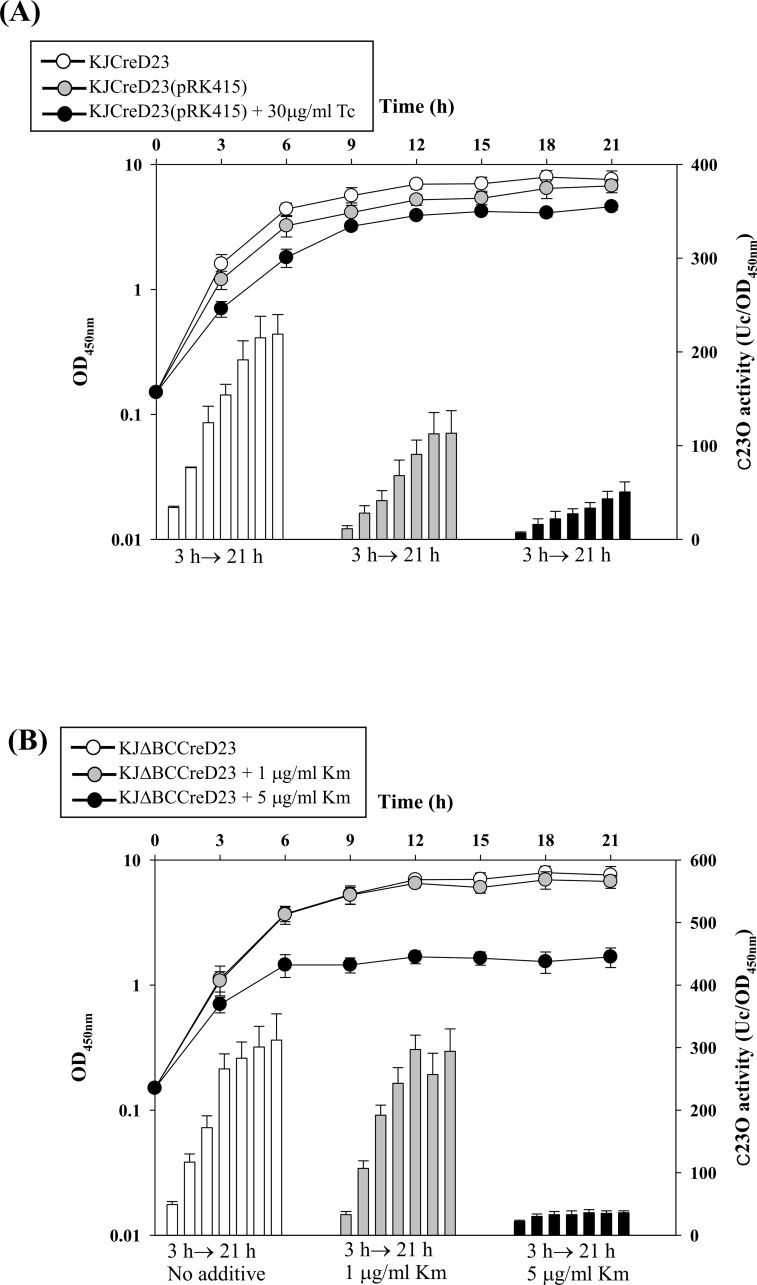
The promoter activity of *creD* gene was regulated by the bacterial culture density. (A) The impact of plasmid and tetracycline on the bacterial growth and C23O expression of strain KJCreD23. Plasmid pRK415 was transported into KJCreD23 by conjugation. The bacterial growth (by recording the OD_450nm_) and C23O activity expressed from KJCreD23, KJCreD23(pRK415), and KJCreD23(pRK415) with 30 μg/ml tetracycline were monitored every 3 h. (B) The impact of kanamycin (Km) on the bacterial growth and C23O expression of strain KJΔBCCreD23. The bacterial growth (by recording the OD_450nm_) and C23O activity of KJΔBCCreD23 in the absence and presence of kanamycin (1 or 5 μg/ml) were monitored every 3 h. (C) The impact of menadione (K3) on the bacterial growth and C23O expression of strain KJΔBCCreD23. The bacterial growth (by recording the OD_450nm_) and C23O activity of KJΔBCCreD23 in the absence and presence of K3 (2 or 30 μg/ml) were monitored every 3 h. (D) The impact of benzalkonium chloride (BC) on the bacterial growth and C23O expression of strain KJΔBCCreD23. The bacterial growth (by recording the OD_450nm_) and C23O activity of KJΔBCCreD23 in the absence and presence of BC (1 or 5 μg/ml) were monitored every 3 h.

Given the inconsistency of the aforementioned results of the plasmid transcription fusion construct (KJ(pCreD_xylE_)) and the chromosomal transcription fusion construct (KJCreD23) (S2 Fig), we wondered whether the presence of the plasmid or the addition of tetracycline for plasmid maintenance during plasmid-borne promoter assay caused the bias. We compared the C23O activities of KJCreD23(pRK415) in the absence and presence of tetracycline (30 μg/ml) at a concentration to maintain the plasmid pRK415. The presence of plasmid pRK415 and the addition of tetracycline compromised bacterial growth and *creD* expression was attenuated (Fig 3A). Furthermore, the degree of attenuation of *creD* expression was correlated with the decrease in bacterial growth. Therefore, we assumed that the *P*_*creD*_ activity is in response to the culture density. To test this, we determined the bacterial growth and the *P*_*creD*_ promoter activity in different conditions. To avoid the impact of CreBC TCS on the *P*_*creD*_ activity, we determined the C23O activities expressed from KJΔBCCreD23, which represents the *P*_*creD*_ activity without the influence of the CreBC TCS. The KJΔBCCreD23 cells were inoculated into fresh LB at an initial OD_450nm_ of 0.15. The cells were exposed to stressors and the cell growth (OD_450nm_) and *P*_*creD*_ activity (C23O activity) were simultaneously recorded. The tested stresses included antibiotics (kanamycin), oxidative stress (menadione), and detergent (benzalkonium chloride). We categorized the outcome of the stress tests as either affecting bacterial growth or not. As shown in Fig 3B–3D, a reduced *P*_*creD*_ activity was associated with a corresponding decrease in culture density. These results suggest that this phenotype is not a consequence of stress but is due to the decrease in bacterial culture density. If the stresses minimally affect the bacterial culture density, the C23O activity expressed by KJΔBCCreD23 is as high as that of the non-treated culture. This observation provides a reasonable explanation for the aforementioned discrepancy in the promoter activity assay between strains KJCreD23 and KJ(pCreD_xylE_) (S2 Fig). In the plasmid transcription fusion assay system KJ(pCreD_xylE_), the plasmid and tetracycline compromise the bacterial growth and influence the *P*_*creD*_ activity. The advantage of using the chromosomal transcription fusion constructs is that the plasmids and tetracycline are avoided.

### *P*_*creD*_ activity is not affected in the presence of phosphor-mimic variant of CreB, CreB(D55E)

**Fig 4:**
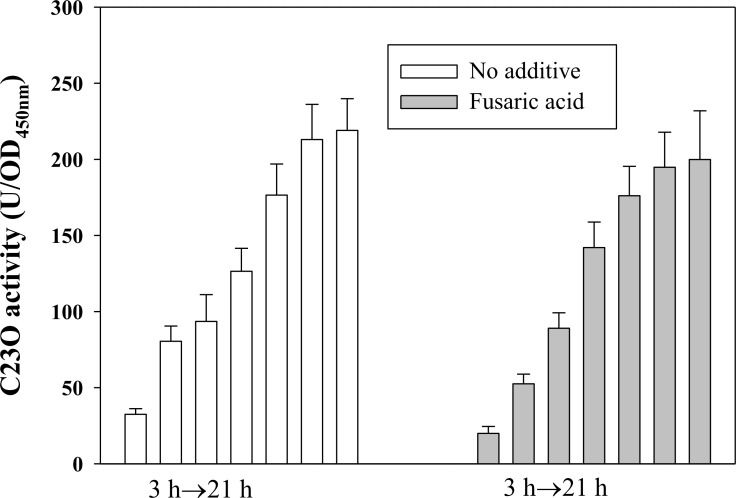
The impact of phosphor-mimic variant of CreB, CreB(D55E), on the promoter activity of *creD* gene. Overnight culture of strain KJCreD23Fua::CreB(D55E) was inoculated into the fresh medium to the OD_450_ of 0.15 in the absence and presence of fusaric acid (30 μg/ml). Cells were grown aerobically and the C23O activity were measured every 3 h.

Given the evidence that the loss of function of CreBC increases the *creD* expression in wild-type KJ (Fig 2), we next tested whether extra activated CreB can affect the expression of *creD*. In other microorganisms, a mutation converting the conserved aspartate to glutamate at the site of phosphorylation constitutively activates the response regulator [21]. Since the stimuli for CreBC activation is unknown, we generated the phosphor-mimic variant of CreB, CreB(D55E), by site-directed mutagenesis. To determine the effect of CreB(D55E) on *creD* expression, strain KJCreD23Fua::CreB(D55E) was constructed in which *creB(D55E)* is inducibly expressed by fusaric acid treatment. First, we assessed the concentration of fusaric acid at which the expression of *creB(D55E)* can be triggered, but bacterial growth is minimally affected. We found that 30 μg/ml of fusaric acid is ideal (S3 Fig). The growth and C23O activities of KJCreD23Fua::CreB(D55E) were comparatively determined in the absence and presence of fusaric acid (30 μg/ml). Fig 4 demonstrates that the C23O activity expressed by KJCreD23Fua::CreB(D55E) was not affected in the presence of fusaric acid, although it was slightly attenuated at the time points of 3 and 6 h.

### *P*_*creD*_ activity is minimally affected by β-lactams challenge

**Fig 5:**
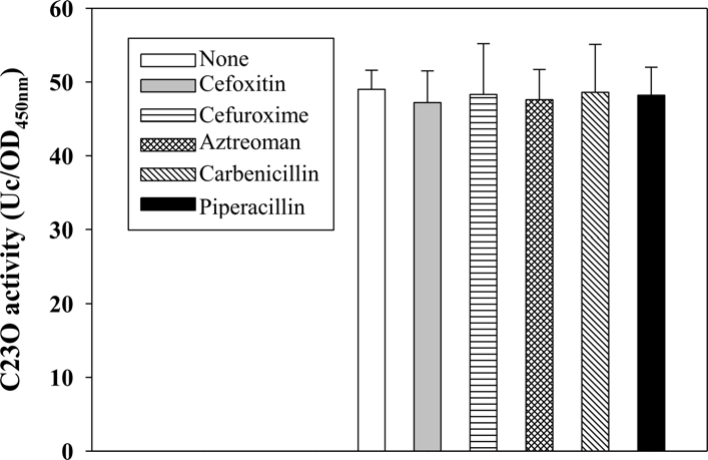
The impact of β-lactam on the promoter activity of *creD* gene. Overnight culture of KJΔBCCreD23 was inoculated into the fresh medium to the OD_450_ of 0.15. After 30-min culture, the β-lactam as indicated was added and the culture was further incubated for 3 h. The OD_450_ and C23O activity were measured.

Zamorano *et al*. report that the presence of β-lactams (such as imipenem or cefoxitin), which can interact with PBP4, activate the CreBC system and increase *creD* expression in *P*. *aeruginosa* [8]. Herein, we investigated whether β-lactams affect the promoter activity of *P*_*creD*_. We tested the effect of 50 μg/ml of β-lactam and found no influence on bacterial growth. The presence of β-lactams did not affect the C23O activity of KJΔBCCreD23 (Fig 5).

### *CreD* deletion mutant displayed a filamentous morphology

The deletion mutant, KJΔCreD, and the complementary strain, KJΔCreD(pCreD), were used to determine the physiologic significance of CreD.

First, the growth of KJ, KJΔCreD, and KJΔCreD(pCreD) was assessed by monitoring the OD_450nm_ every 3 h. The strains had the same growth rates at 30°C, 37°C, and 40°C (S4 Fig).

**Fig 6:**
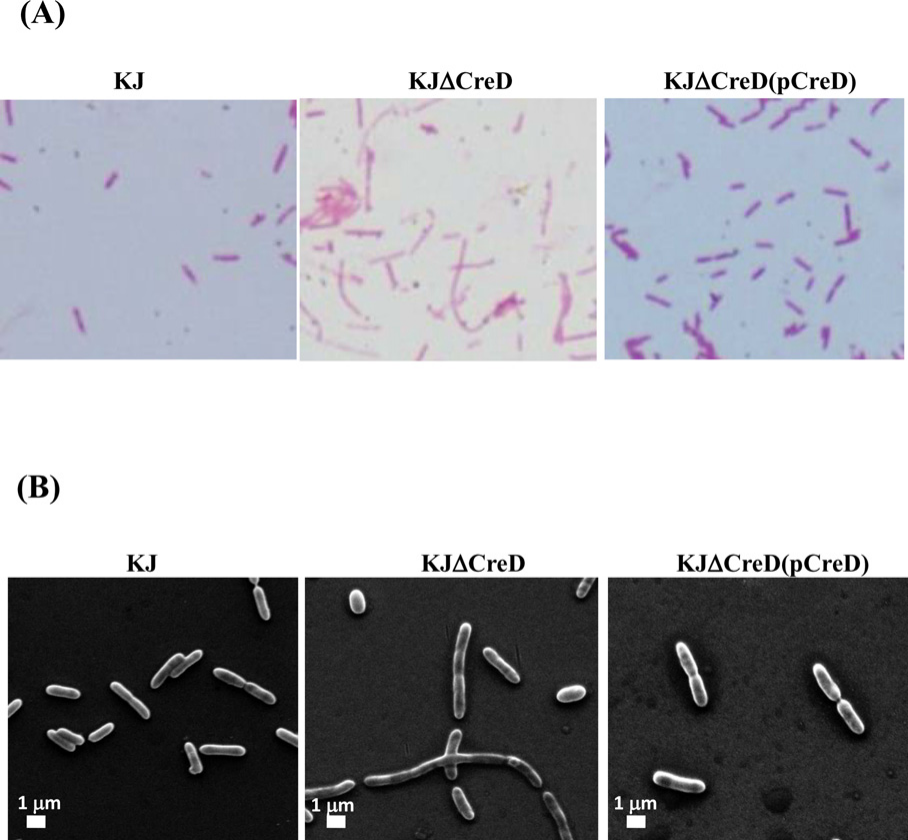
The impact of CreD on bacterial morphology. (A) Bacterial cells were stained with Gram stain and examined by light microscopy. (B) Scanning electron microscopy was performed as described in Materials and Methods. Images are representative of different fields of bacteria from exponentially growing cultures at 37°C.

The effect of *creD* inactivation on cell morphology was examined by light microscopy and SEM. Inactivation of *creD* led to a striking phenotype of filamentous cells. In KJΔCreD cells, constriction appeared to begin at the septal ring but could not be completed, indicating a defect in constriction and separation. However, a fraction of KJΔCreD cells still seemed to maintain morphology similar to that of wild-type KJ cells. The aberrations in the morphology of KJΔCreD could be largely restored by complementation with an intact *creD* gene (Fig 6).

### Cell envelope integrity of the *creD* deletion mutant was compromised

**Fig 7:**
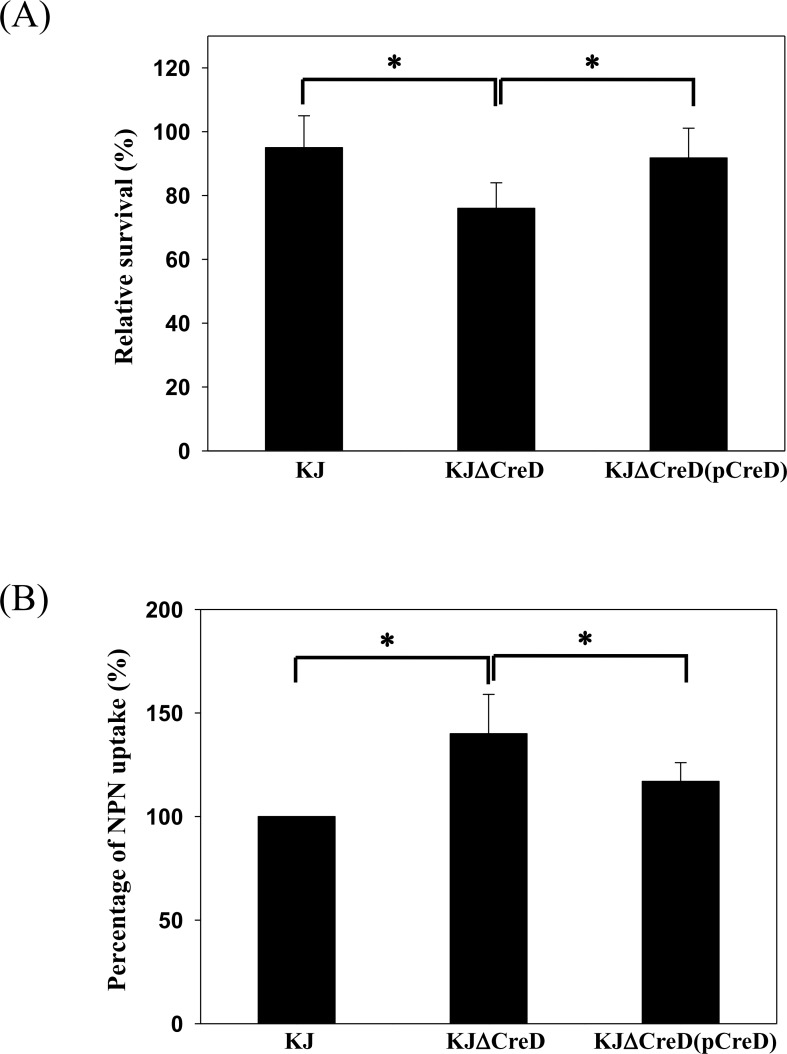
The impact of CreD on cell envelope integrity. Each bar represents the mean of three independent experiments. Error bars indicate the average deviation. *, *p≤0*.*05* significance calculated by s Student’s *t*-test. (A) Sodium dodecyl sulfate (SDS) survival analysis. The survival of KJ, KJΔCreD, and KJΔCreD(pCreD) in LB broth without or with 0.01% SDS was determined by colony forming units (CFUs) counting. The percentage of survival was defined as the CFUs ratio of the SDS-additive group to the SDS-free counterpart. (B) N-phenylanphthylamine (NPN) uptake assay. Each microtiter well was inoculated with 100 μl of the OD_450_ 0.5 bacterial culture and 15 μM NPN, and incubated for 5 min. Fluorescence was monitored by fluorescence spectrophotometer at excitation and emission wavelengths of 355 nm and 402 nm, respectively.

Given that inactivation of *creD* caused alterations in morphology, it seems probable that CreD can participate in the maintenance of cell envelope integrity. The cell envelope integrity of KJΔCreD cells was therefore assessed by an SDS sensitivity assay. Upon 5-min challenge with 0.01% SDS, the survival rate of KJΔCreD was found to be lower than that of wild-type KJ, and the complemented strain, KJΔCreD(pCreD), showed restoration of the survival rate (Fig 7A). Subsequently, the outer membrane permeability of KJΔCreD cells was assessed with the N-phenyl-1-napthylamine (NPN) uptake assay and the susceptibility test for vancomycin. KJΔCreD cells had a higher tendency for NPN uptake than wild-type KJ, and this defect could be partially restored by complementation with a *creD* gene (Fig 7B), indicating that the outer membrane of KJΔCreD cells had a higher tendency for cationic compound uptake than the outer membrane of wild-type KJ. In addition, the outer membrane of gram-negative bacteria is generally a barrier for high-molecular-weight antibiotics such as vancomycin. Therefore, vancomycin susceptibility can be used as an indicator for evaluating the outer membrane permeability of gram-negative bacteria for high-molecular-weight substances. The susceptibility of KJ, KJΔCreD, and KJΔCreD(pCreD) to vancomycin was tested by the E-test. The MICs of KJ, KJΔCreD, and KJΔCreD(pCreD) cells for vancomycin were >256, 128, and >256 μg/ml, respectively (detection limit of the E-test strip for vancomycin, 256 μg/ml). Collectively, this evidence leads to the conclusion that CreD plays a critical role in envelope homeostasis. Loss of CreD increased the membrane susceptibility to SDS and altered the outer membrane permeability, which increased the outer membrane uptake efficiency for cationic compounds and permeability for high-molecular-weight substances.

### Inactivation of *creD* triggered the σ^E^-mediated envelope stress response

**Fig 8:**
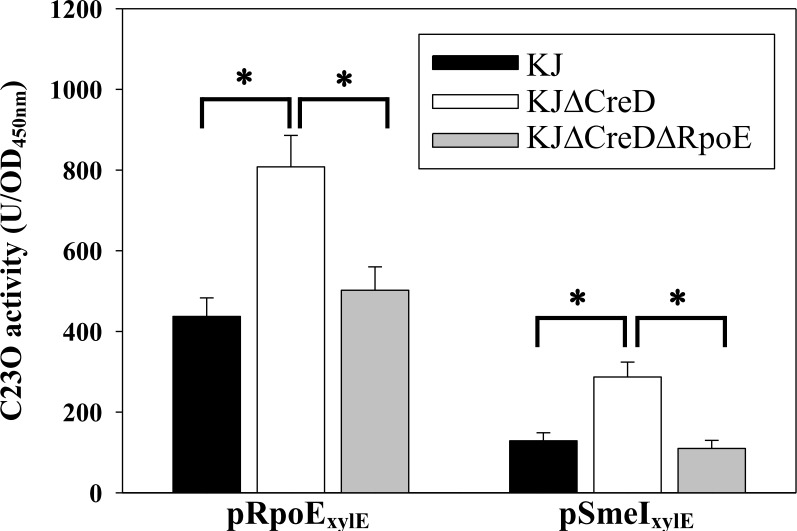
The linkage between *creD* inactivation and σ^E^-mediated envelope stress response. The promoter transcriptional fusion constructs of *rpoE* and *smeI* genes, pRpoE_xylE_ and pSmeI_xylE_, were transferred into KJ, KJΔCreD, and KJΔCreDΔRpoE cells and the expressed C23O activities were determined, respectively. Each bar represents the mean of three independent experiments. Error bars indicate the average deviation. *, *p*≤0.01 significance calculated by s Student’s *t*-test.

When the cell envelope integrity is compromised, bacterial cells generally trigger a variety of envelope stress response (ESRs) to alleviate the envelope stresses. Of the known ESRs, σ^E^-mediated ESR is the most common mechanism in gram-negative bacteria [22]. Recently, σ^E^-mediated ESR has been reported for *S*. *maltophilia*; it was found that σ^E^ itself and the *smeIJK* operon are members of the σ^E^ regulon [18]. Considering the effect of *creD* inactivation on cell envelope integrity and outer membrane permeability (Fig 7), we speculated whether loss of CreD induces activation of the σ^E^ pathway. To determine this, the expression of σ^E^ and the *smeIJK* operon for wild-type KJ and KJΔCreD was compared using the promoter transcription fusion assay. As shown in Fig 8, deletion of *creD* increased the expression of σ^E^ and *smeIJK* by a factor of 1.85-fold and 2.23-fold, respectively, and this upregulation was reverted when *rpoE* was inactivated, supporting that the loss of the inner membrane protein CreD triggers σ^E^-mediated ESR in *S*. *maltophilia*.

## Discussion

S1 Fig shows the comparisons of *creBCD* clusters between *S*. *maltophilia*, *E*. *coli*, *P*. *aeruginosa*, and *Aeromonas* spp. *CreD* of *E*. *coli* is part of the *creABCD* operon and has its own promoter [23]. The *creD* of *S*. *maltophilia* and *Aeromonas* spp. is a one-gene transcript. In this study, we investigated the regulatory role of the *creBC* TCS in *creD* expression. *CreD* of *S*. *maltophilia* is downregulated by CreBC, since *creD* expression increases when *creBC* is inactivated (Fig 2), which is not consistent with the *P*. *aeruginosa* model in which deletion of *creBC* does not change *creD* expression [5]. These results indicate that the CreBC system of *S*. *maltophilia* is active in laboratory cultured condition.

Since the exact stimuli for CreBC activation of *S*. *maltophilia* are unknown, we used an overexpressing phosphor-mimic variant of CreB, CreB(D55E), to monitor the effect of CreBC activation on *creD* expression. We found no significant alteration in the *creD* expression when CreB(D55E) is overexpressed (Fig 4), which suggests that either CreB(D55E) is not a functional transcription regulator that affects expression of the CreBC regulon or CreBC TCS is fully activated in the *S*. *maltophilia* isolate KJ and the presence of CreB(D55E) cannot further activate the CreBC TCS. In this second scenario, the activated CreB of *S*. *maltophilia* plays a negative regulatory role in the expression of *creD*, which is opposite of its role in *E*. *coli*, *Aeromonas* spp., and *P*. *aeruginosa* [3–5].

Based on the known regulatory circuit of *creBC* and *creD*, increased expression of *creD* is as an indicator of *creBC* TCS activation in *E*. *coli* and *P*. *aeruginosa* systems [3,5]. However, this is not true in *S*. *maltophilia*. Our results suggest that the expression of *creD* is regulated by CreBC TCS and bacterial culture density. Some intrinsic physiologic responses or external stimuli that are not related to CreBC activation can alter the bacterial growth rate and culture density, which, in turn, affects the *creD* expression. Therefore, the expression of *creD* is not an optimal indicator of *creBC* activation in *S*. *maltophilia*.

The cell envelope of gram-negative bacteria consists of two distinct membranes, the inner membrane (IM) and the outer membrane (OM), which are separated by an aqueous compartment, the periplasm. Approximately a quarter of all genes in the *E*. *coli* genome encode integral inner membrane proteins, whereas the inner membrane lipid bilayer occupies less than 4% of the total bacterial volume [24]. Therefore, the expression of inner membrane proteins should be tightly regulated to maintain envelope integrity. Herein, we demonstrated that CreD is constitutively expressed and that its expression gradually increases in proportion to bacterial culture density (Fig 2). Furthermore, we found that inactivation of *creD* causes morphological aberrations (Fig 6), compromises envelope integrity, and increases outer membrane permeability (Fig 7), which then trigger the σ^E^-mediated envelope stress response (Fig 8). Therefore, we propose two possible roles of CreD in the maintenance of envelope integrity; the two possibilities are not mutually exclusive: (i) CreD appears to act as an architectural frame (a structural component) of the inner membrane and play a critical role in the maintenance of envelope integrity. When the bacterial culture density increases, the expression of CreD is synchronously increased to meet the requirement of cell membrane expansion, thus exquisitely maintaining the balance between bacterial growth and CreD biological function. (ii) CreD may function as a channel for extrusion of noxious metabolites that are produced during bacterial growth. Therefore, the expression of *creD* is proportional to active bacterial growth. If the CreD expression is impaired, the accumulated noxious metabolites may compromise envelope integrity and trigger ESR.

## Supporting Information

S1 Fig Comparisons between *creBCD* cluster in *S*. *maltophilia* and its homologues in *Escherichia coli*, *Pseudomonas aeruginosa*, and *Aeromonas* spp. (TIF)

S2 Fig The C23O activity expressed by KJCreD23 and KJ(pCreD_xylE_). (TIF)

S3 Fig The impact of fusaric acid concentrations on the bacterial growth and *creB* transcript expression of KJCreD23Fus::CreB(D55E). (TIF)

S4 Fig The impact of CreD on the bacterial growth at different temperatures. (TIF)

S1 Table Bacterial strains, plasmids and primers used in this study. (TIF)

## References

[1] Stock AM, Robinson VL, Goudreau PN. Two-component signal transduction. Annu Rev Biochem. 2000; 69: 183–215. 10966457 10.1146/annurev.biochem.69.1.183

[2] Olaitan AO, Morand S, Rolain JM. Mechanisms of polymyxin resistance: acquired and intrinsic resistance in bacteria. Front Microbiol. 2014; 26: 643. 10.3389/fmicb.2014.00643 PMC4244539 25505462

[3] Avison MB, Horton RE, Walsh TR, Bennett PM. *Escherichia coli* CreBC is a global regulator of gene expression that responds to growth in minimal media. J Biol Chem. 2001; 276: 26955–26961. 11350954 10.1074/jbc.M011186200

[4] Tayler AE, Ayala JA, Niumsup P, Westphal K, Baker JA, Zhang L, et al Induction of β-lactamase production in *Aeromonas hydrophila* is responsive to β-lactam-mediated changes in peptidoglycan composition. Microbiology. 2010; 156: 2327–2325. 10.1099/mic.0.035220-0 20430811

[5] Moya B, Dotsch A, Juan C, Blazquez J, Zamorano L, Haussler S, et al β-Lactam resistance response triggered by inactivation of a nonessential penicillin-binding protein. PLoS Pathog. 2009; 5: e1000353 10.1371/journal.ppat.1000353 19325877 PMC2654508

[6] Cariss SJ, Tayler AE, Avison MB. Defining the growth conditions and promoter-proximal DNA sequences required for activation of gene expression by CreBC in *Escherichia coli* . J Bacteriol. 2008; 190: 3930–3939. 10.1128/JB.00108-08 18375564 PMC2395042

[7] Alksne LE, Rasmussen BA. Expression of the AsbA1, OXA-12, and AsbM1 beta-lactamases in *Aeromonas jandaei* AER 14 is coordinated by a two-component regulon. J Bacteriol. 1996; 179: 2006–2013. 10.1128/jb.179.6.2006-2013.1997 PMC178926 9068648

[8] Zamorano L, Moya B, Juan C, Mulet X, Blazquez J, Oliver A. The *Pseudomonas aeruginosa* CreBC two-component system plays a major role in the response to β-lactams, fitness, biofilm growth, and global regulation. Antimicrob Agents Chemother. 2014; 58: 5084–5095. 10.1128/AAC.02556-14 24936599 PMC4135852

[9] Niumsup PA, Simm M, Nurmahomed K, Walsh TR, Bennett PM, Avison MB. Genetic linkage of the penicillinase gene, *amp*, and *blrAB*, encoding the regulator of β-lactamase expression in *Aeromonas* Spp. J Antimicrob Chemother. 2003; 51: 1351–1358. 12746371 10.1093/jac/dkg247

[10] Brooke JS. *Stenotrophomonas maltophilia*: an emerging global opportunistic pathogen. Clin Microbiol Rev. 2012; 25: 2–41. 10.1128/CMR.00019-11 22232370 PMC3255966

[11] Crossman LC, Gould VC, Dow JM, Vernikos GS, Okazaki A, Sebaihia M, et al The complete genome, comparative and functional analysis of *Stenotrophomonas maltophilia* reveals an organism heavily shielded by drug resistance determinants. Genome Biol. 2008; 17: R74. 10.1186/gb-2008-9-4-r74 PMC2643945 18419807

[12] Li XZ, Zhang L, Poole K. SmeC, an outer membrane multidrug efflux protein of *Stenotrophomonas maltophilia* . Antimicrob Agents Chemother. 2002; 46: 333–343. 11796339 10.1128/AAC.46.2.333-343.2002 PMC127032

[13] Lin CW, Lin HC, Huang YW, Chung TC, Yang TC. Inactivation of *mrcA* gene derepresses the basal-level expression of L1 and L2 β-lactamases in *Stenotrophomonas maltophilia* . J Antimicrob Chemother. 2011; 66: 2033–2037. 10.1093/jac/dkr276 21719470

[14] Huang YW, Wu CJ, Hu RM, Lin YT, Yang TC. An interplay among membrane-bound lytic transglycosylase D1, CreBC two-component regulatory system, AmpNG-AmpDI-NagZ-AmpR regulatory circuit, and L1/L2 β-lactamases expression in *Stenotrophomonas maltophilia* . Antimicrob Agents Chemother. 2015 (Accepted). 10.1128/AAC.05179-14 PMC4604389 26282431

[15] Hu RM, Huang KJ, Wu LT, Hsiao YJ, Yang TC. Induction of L1 and L2 β-lactamases of *Stenotrophomonas maltophilia* . Antimicrob Agents Chemother. 2008; 52: 1198–1200. 18086856 10.1128/AAC.00682-07 PMC2258547

[16] Yang TC, Huang YW, Hu RM, Huang SC, Lin YT. AmpD_I_ is involved in expression of the chromosomal L1 and L2 β-lactamases of *Stenotrophomonas maltophilia* . Antimicrob Agents Chemother. 2009; 53: 2902–2907. 10.1128/AAC.01513-08 19414581 PMC2704650

[17] Livak KJ, Schmittgen TD. Analysis of relative gene expression data using real-time quantitative PCR and the 2-(Delta DeltaC(T)) Method. Methods. 2001; 25: 402–408. 11846609 10.1006/meth.2001.1262

[18] Huang YW, Liou RS, Lin YT, Huang HH, Yang TC. A linkage between SmeIJK efflux pump, cell envelope integrity, and σ^E^-mediated envelope stress response in *Stenotrophomonas maltophilia* . PLoS ONE. 2014; 9: e111784 10.1371/journal.pone.0111784 25390933 PMC4229105

[19] Lin CW, Huang YW, Hu RM, Chiang KH, Yang TC. The role of AmpR in the regulation of L1 and L2 β-lactamases in *Stenotrophomonas maltophilia* . Res Microbiol. 2009; 160: 152–158. 10.1016/j.resmic.2008.11.001 19071216

[20] Hu RM, Liao ST, Huang CC, Huang YW, Yang TC. An inducible fusaric acid tripartitle efflux pump contributes to the fusaric acid resistance in *Stenotrophomonas maltophilia* . PLoS ONE. 2012; 7: e51053 10.1371/journal.pone.0051053 23236431 PMC3517613

[21] Klose KE, Weiss DS, Kustu S. Glutamate at the site of phosphorylation of nitrogen-regulatory protein NTRC mimics aspartyl-phosphate and activates the protein. J Mol Biol. 1993; 232:67–78. 8331671 10.1006/jmbi.1993.1370

[22] Hayden JD, Ades SE. The extracytoplasmic stress factor, σE, is required to maintain cell envelope integrity in *Escherichia coli* . PLoS ONE. 2008; 3: e1573 10.1371/journal.pone.0001573 18253509 PMC2215328

[23] Drury LS, Buxton RS. Identification and sequencing of the *Escherichia coli* cet gene which codes for an inner membrane protein, mutation of which causes tolerance to colicin E2. Mol Microbiol. 1998; 2: 109–119. 10.1111/j.1365-2958.1988.tb00012.x 2835585

[24] Elofsson A, von Heijne G. Membrane protein structure: prediction versus reality. Annu Rev Biochem. 2007; 76: 125–140. 17579561 10.1146/annurev.biochem.76.052705.163539

