# Supplementary figures and images for: Expression and Functions of CreD, an Inner Membrane Protein in Stenotrophomonas maltophilia
Source: PLoS One. 2015 Dec 23;10(12):e0145009. doi: 10.1371/journal.pone.0145009 (PMC4689548; doi:10.1371/journal.pone.0145009)

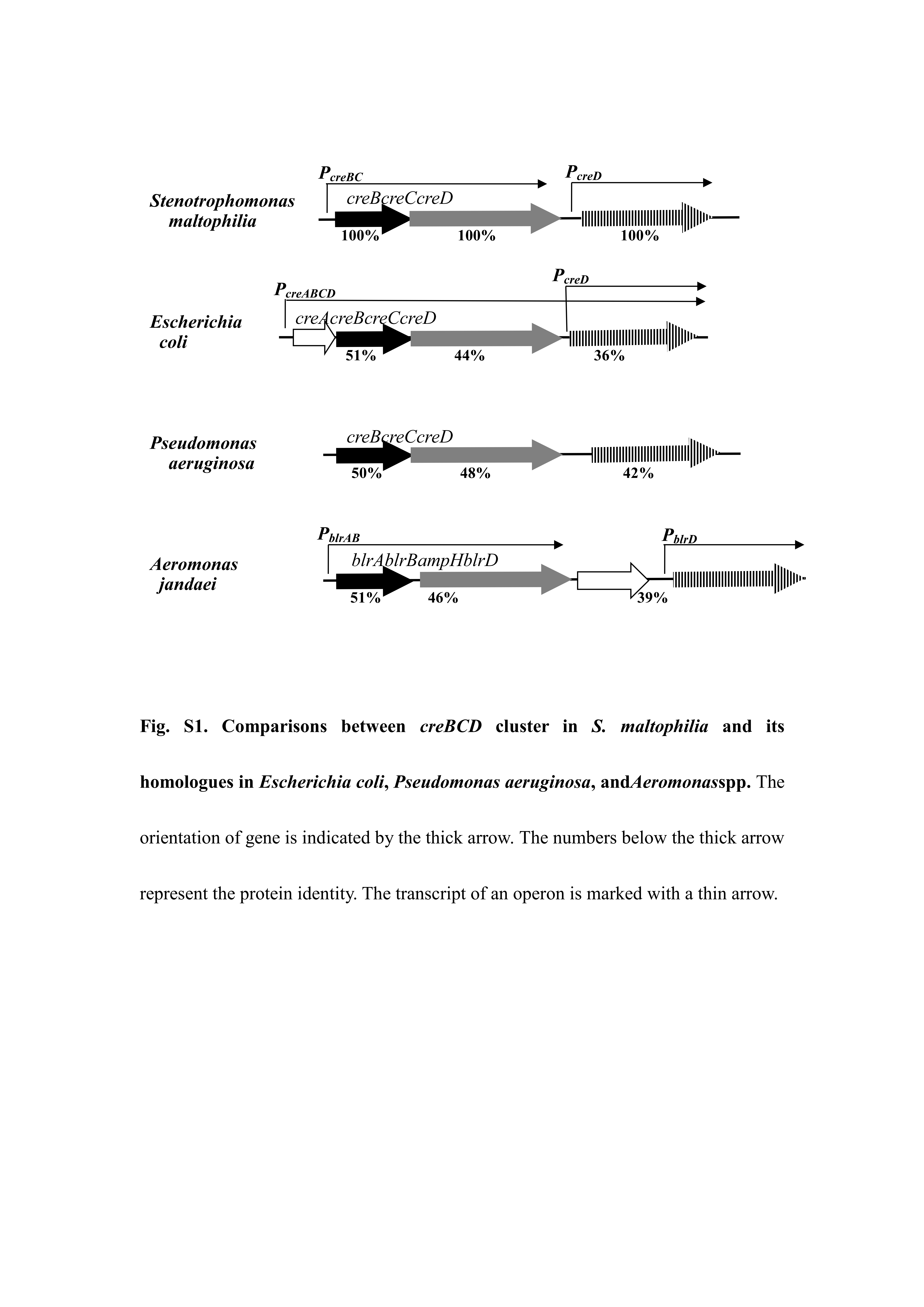

Supplement: S1 Fig — (TIF) [file pone.0145009.s001.tif]

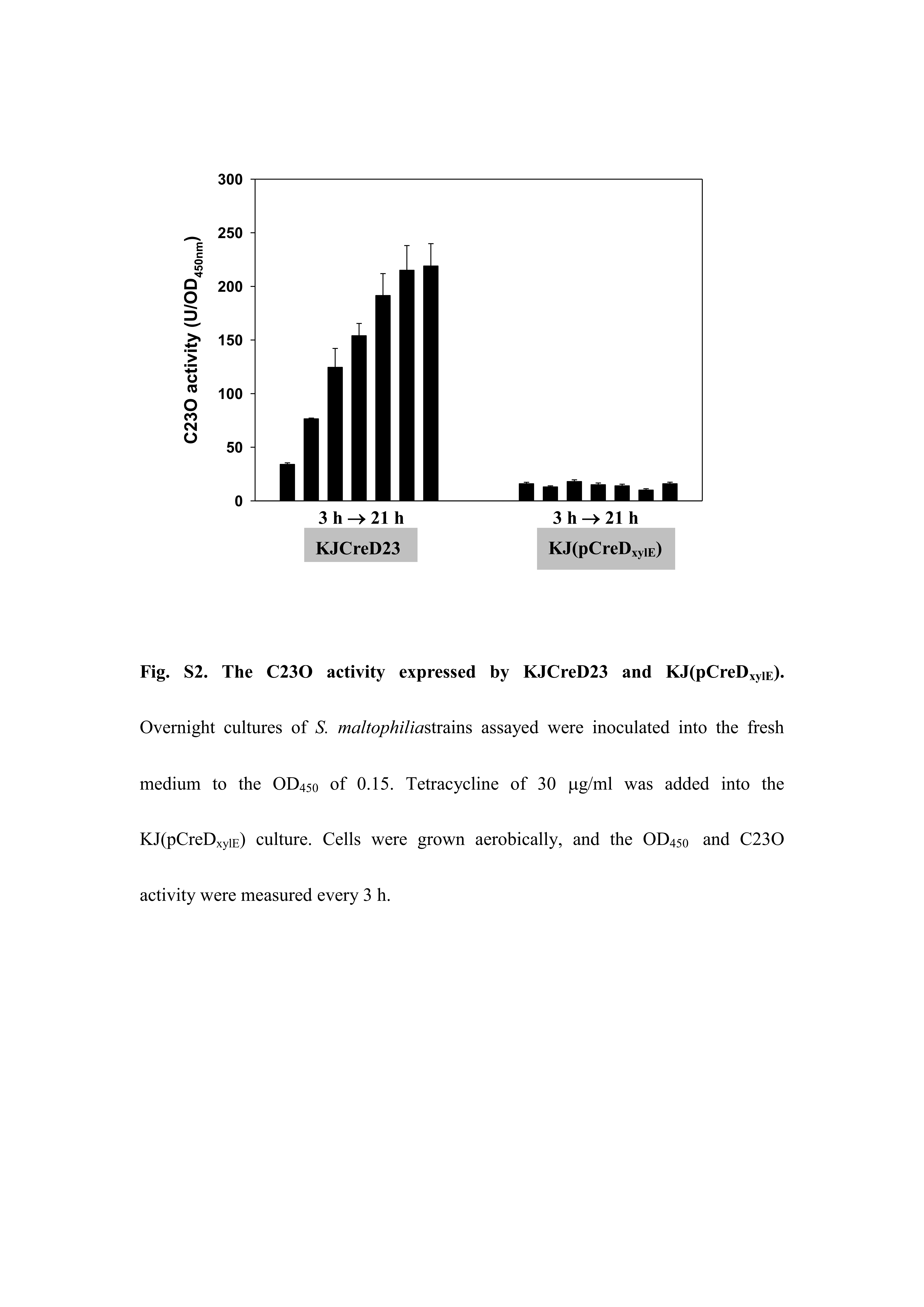

Supplement: S2 Fig — (TIF) [file pone.0145009.s002.tif]

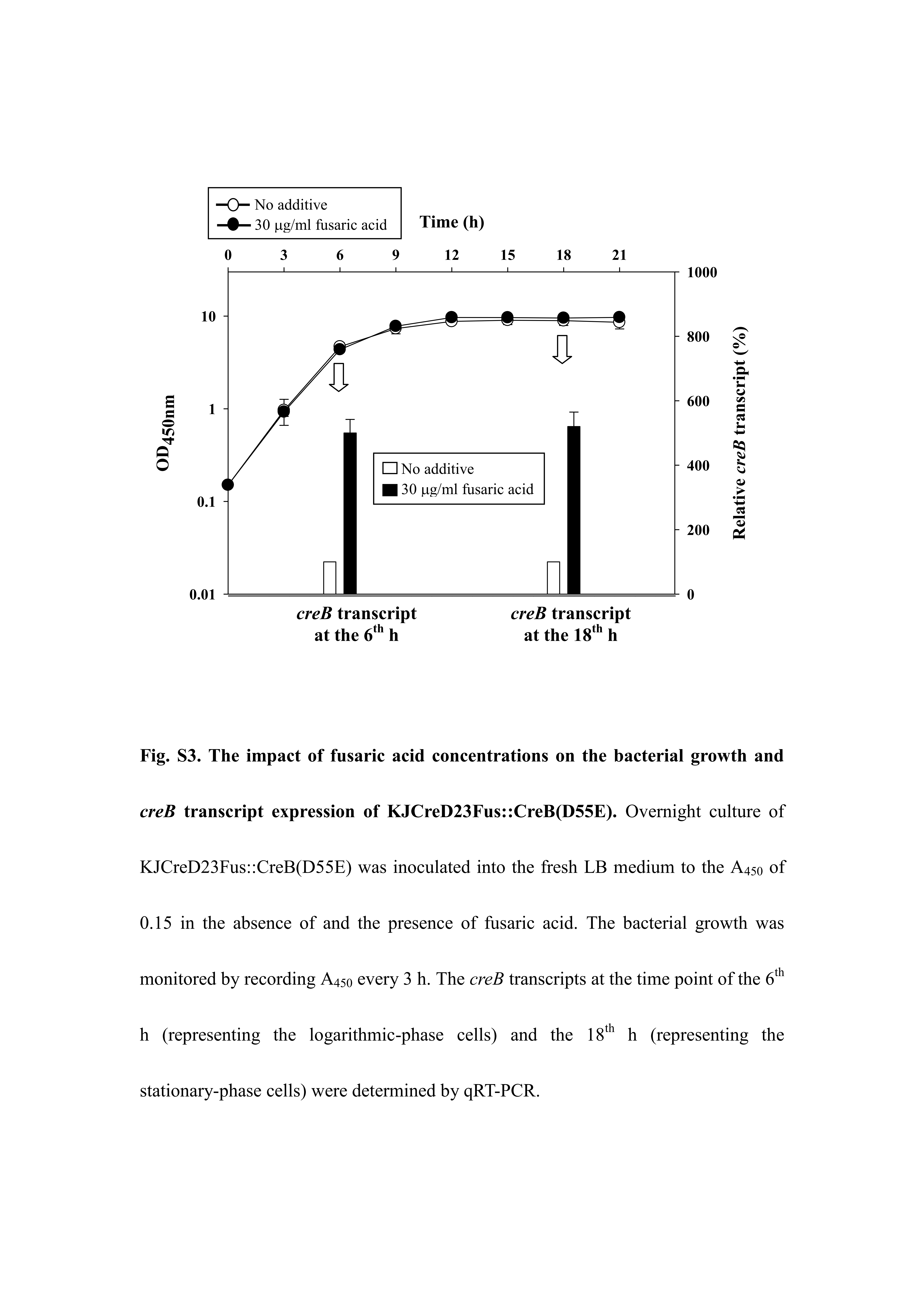

Supplement: S3 Fig — (TIF) [file pone.0145009.s003.tif]

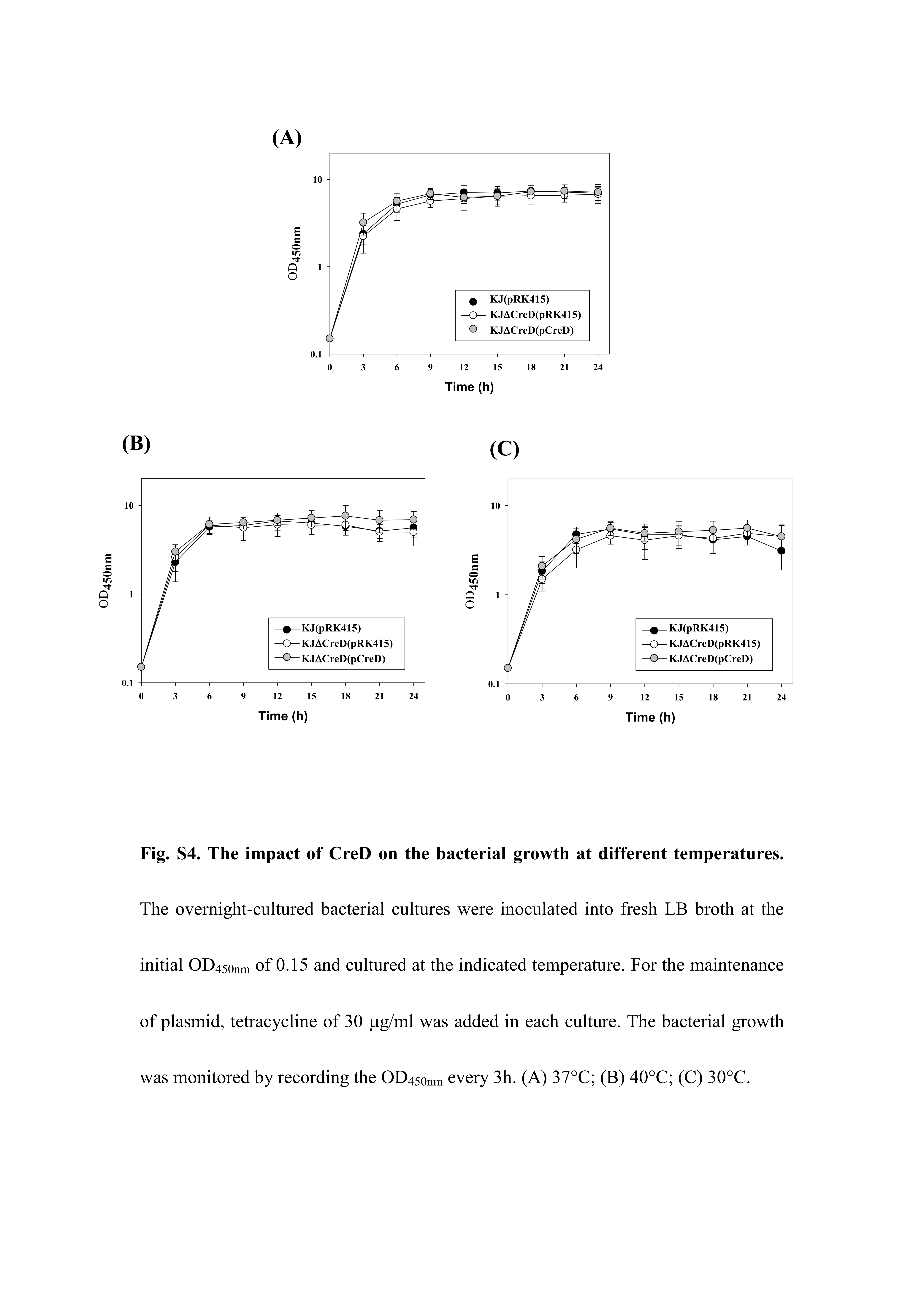

Supplement: S4 Fig — (TIF) [file pone.0145009.s004.tif]

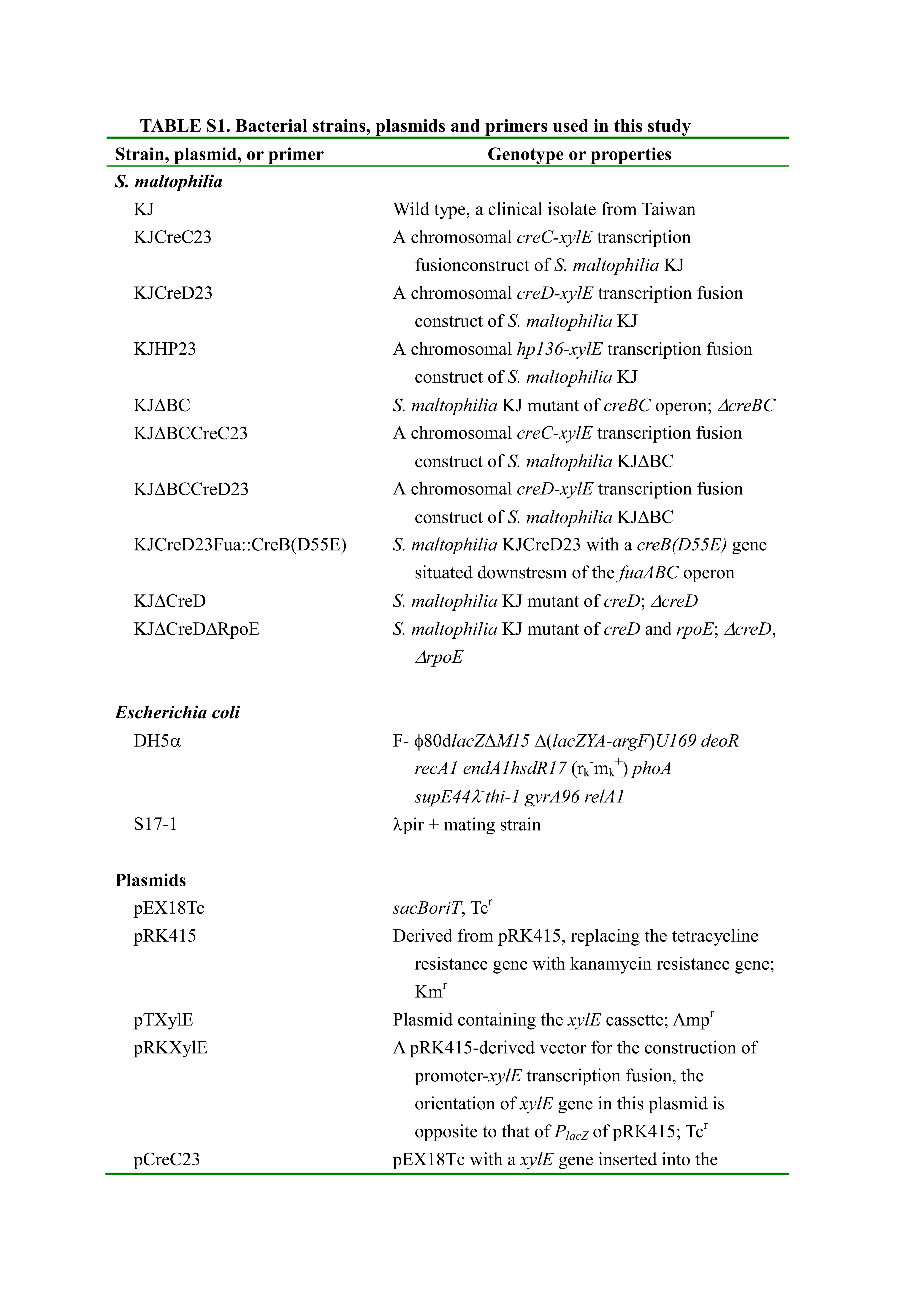

Supplement: S1 Table — (TIF) [file pone.0145009.s005.tif]
